# Changing trends in the microvascular reconstruction and oral rehabilitation following maxillary cancer

**DOI:** 10.1007/s00405-022-07277-y

**Published:** 2022-02-01

**Authors:** Simon N. Rogers, Ashni Adatia, Stephanie Hackett, Angela Boscarino, Anika Patel, Derek Lowe, Christopher J. Butterworth

**Affiliations:** 1 Regional Maxillofacial Unit, Liverpool Head and Neck Centre, Liverpool University Hospital NHS Foundation Trust, Lower Lane, Liverpool, UK; 2 Faculty of Health and Life Sciences, University of Liverpool, Liverpool, UK; 3 Astraglobe Ltd, Congleton, Cheshire, England; 4 Faculty of Health, Social Care and Medicine, Edge Hill University, Ormskirk, L39 4QP England

**Keywords:** Maxillectomy, Free tissue transfer, Reconstruction, Oral rehabilitation, Zygomatic implants, ZIP flap

## Abstract

**Purpose:**

The maxillectomy defect is complex and the best means to achieve optimal reconstruction, and dental rehabilitation is a source of debate. The refinements in zygomatic implant techniques have altered the means and speed by which rehabilitation can be achieved and has also influenced the choice regarding ideal flap reconstruction. The aim of this study is to report on how the method of reconstruction and oral rehabilitation of the maxilla has changed since 1994 in our Institution, and to reflect on case mix and survival.

**Methods:**

Consecutive head and neck oncology cases involving maxillary resections over a 27-year period between January 1994 and November 2020 were identified from hospital records and previous studies. Case note review focussed on clinical characteristics, reconstruction, prosthetic rehabilitation, and survival.

**Results:**

There were 186 patients and the tumour sites were: alveolus for 56% (104), hard palate for 19% (35), maxillary sinus for 18% (34) and nasal for 7% (13). 52% (97) were Brown class 2 defects. Forty-five patients were managed by obturation and 78% (142/183) had free tissue transfer. The main flaps used were radial (52), anterolateral thigh (27), DCIA (22), scapula (13) and fibula (11). There were significant changes over time regarding reconstruction type, use of primary implants, type of dental restoration, and length of hospital stay. Overall survival after 24 months was 64% (SE 4%) and after 60 months was 42% (SE 4%).

**Conclusion:**

These data reflect a shift in the reconstruction of the maxillary defect afforded by the utilisation of zygomatic implants.

## Introduction

Oncological resection of the maxilla results in a complex defect which can be a challenge to optimally reconstruct and provide dental rehabilitation and function in the context of personalised treatment planning and outcomes. There are several reconstructive options including local tissue flaps, prosthetic obturation, the use of osseointegrated implants, as well as soft tissue and composite free tissue transfer [1, 2]. Many factors influence the most appropriate reconstruction such as the extent of the defect, dental status, motivation of the patient for oral rehabilitation, comorbidity, institutional experience and clinician preferences. The complexity of the defect in part is reflected in the various classifications that have been previously advocated [3–9] and the options for reconstruction are epitomised by the different algorithms and techniques published over the years [3–5, 10–15]. The reconstruction of the defect might be influenced to a degree, by the relatively poor survival prognosis in those having maxillary resection compared to other head and neck cancer sites, notably oral cavity [16, 17]. This leads to a concept of radical palliative surgery with curative intent, such that for those where cure is ultimately not possible there should ideally be a time of comparatively satisfactory function and quality of life associated with minimal treatment burden and necessity for further hospital procedures. In long-term survivors who are recurrence free, secondary oral rehabilitation adds another level of difficulty with the balance of risk versus benefit especially relevant for those who have had post-operative radiotherapy. In this group the risk of osteoradionecrosis and prosthetic failure versus improvement in appearance, chewing, speech and swallowing must be carefully considered [18]. Many patients do not complete full oral rehabilitation and cope and adapt to their outcome [19].

Given the complexity associated with the maxillary defect, the aim of this study is to report changes in the method of reconstruction and oral rehabilitation of the maxilla since 1994 in our Institution, and to reflect on case mix and survival. For the purpose of this study, there is an emphasis on oral rehabilitation; hence, the maxillary defect has focussed on those resections involving the maxillary alveolus, hard palate, maxillary sinus and nasal complex restricted to Classes 1 to 4 of the Brown classification [7].

## Methods

Consecutive head and neck oncology cases involving maxillary resections over a 27-year period between January 1994 and November 2020 at the Liverpool University Foundation NHS Trust were identified from the hospital operating theatre database. The following theatre codes [20] were used to screened eligibility for possible maxillary resection; V06.9 unspecified excision of maxilla, V07.2 partial excision of bone of face, V07.1 extensive excision of bone of face and V07.8 other specified excision of bone of face. Hard palate and maxillary alveolus were considered as part of maxillary resection. Patients were excluded if maxillary surgery had been flagged up in the non-oncology setting, such as osteotomies, temporomandibular surgery, or trauma. Cases with a Brown’s classification of 5 or 6 were also excluded as the focus of the sample were those defects suitable for oral rehabilitation. Datasets from previous research [21, 22] were also searched for cross reference and verified on the electronic hospital records to add cases not identified from the operating theatre database.

Patient data were collected from the electronic case notes, including outpatient clinical letters, multi-disciplinary entries and radiographs. Rehabilitation data were collected from the Liverpool University Dental Hospital Implant Database. Patient clinical and demographic characteristics included the American Society of Anaesthesiologists (ASA) grade [23] site and diagnosis of tumour, operation date, length of stay, Brown’s classification of maxillary resection [7], pathological staging (pTNM) [24], type of flap used for reconstruction, oral rehabilitation, post-operative radio/chemotherapy and survival time.

### Ethical approval

The data were collected as part of the clinical audit process and this study was approved by Liverpool University Hospital NHS Foundation Trust Audit Department (CAMS reference 10074).

### Statistical analysis

Fisher’s exact test was used to compare categorical data between patient subgroups and the Kruskal–Wallis test was used to compare numerical data. The three time periods analysed were tertiles derived from the number of patients each year over the whole time period. Survival statistics were calculated using the Kaplan–Meier method and the log-rank test. SPSS v25 and STATA v13 were used for the analyses. Given the number of tests, statistical significance was interpreted at the 1% level. A small number of patients were operated on more than once during the study period (6 twice and 1 thrice) and these subsequent operations were excluded from the statistical analysis.

## Results

The study sample comprised 186 patients with Brown’s 1–4 maxillary resection seen between 1994 and 2020. Median (IQR) age at primary tumour diagnosis was 69 (59–77) years and 51% (94) were male. Male patients tended to be younger with 23% (22/94) of men aged ≥ 75 years compared to 45% (41/92) of women. Primary tumour site was alveolus for 56% (104), hard palate for 19% (35), maxillary sinus for 18% (34) and nasal for 7% (13). Most (86%, 160) tumours were diagnosed as SCC, 76% (130/172) were pT stage 3–4 and 24% (42/172) were pN positive, with 77% (133/172) being overall stage 3–4. Brown’s classification was level 1 for 26% (48), level 2 for 52% (97), level 3 for 7% (13) and level 4 for 15% (28). ASA was grade 3–4 for 20% (36/179), with 33 grade 3 and 3 grade 4. Surgery alone was used for 40% (75) while for 60% (111) treatment comprised surgery and either radiotherapy, chemotherapy or both. Reconstructive flaps were used for 76% (142), comprising 81 soft flaps and 61 involving hard tissue composite flaps. The main free flaps used were radial (52), anterolateral (27), DCIA (23), scapula (14) and fibula (11). Median (IQR) length of hospital stay (LOS) was 10 (7–16) days.

A fuller breakdown of patient casemix is shown in Table 1, with results also stratified by time period (tertiles), i.e. for 1994–2009, 2010–2015 and 2016–2020. There were significant changes over time in regard to flap reconstruction, LOS, ASA grade and diagnosis. The percentage having flap reconstruction rose from 72% (1994–2009) and 63% (2010–2015) to 91% (2016–2020); the percentages having radial flap reconstructions were 19%, 11% and 51% while the percentages having DCIA flaps were 17%, 17% and 3%. Unsurprisingly the LOS fell over the whole study period, from a median of 15 days (1994–2009) to 10 days (2010–2015) and 9 days (2016–2020). The percentages with ASA grades 3–4 were similar over the 3 time periods, the differences being in the mix of grades 1 and 2 with 18% grade 1’s for 1994–2009, 7% for 2010–2015 and 35% for 2016–2020. All but one of the non-SCC tumours in the study sample were after 2010.

**Table 1:** Casemix, 1994–2020

	1994–2009		2010–2015		2016–2020		*P* value*	Total	
	*N*	%	*N*	%	*N*	%		*N*	%
All patients	54	100	63	100	69	100		186	100
Gender									
Male	22	41	35	56	37	54	0.24	94	51
Female	32	59	28	44	32	46		92	49
Age (operation)									
Median (IQR) years	70 (58–78)		68 (56–78)		71 (63–76)		0.62	69 (59–77)	
Site									
Alveolus	27	50	32	51	45	65	0.06	104	56
Hard palate	14	26	16	25	5	7		35	19
Max sinus	11	20	10	16	13	19		34	18
Nasal	2	4	5	8	6	9		13	7
Overall P stage									
Early (1–2)	7	14	14	25	18	26	0.26	39	23
Late (3–4)	42	86	41	75	50	74		133	77
NK	5		8		1			14	
Diagnosis									
SCC	53	98	51	81	56	81	0.003	160	86
Non-SCC***	1	2	12	19	13	19		26	14
Browns classification									
1	10	19	25	40	13	19	0.04	48	26
2	32	59	23	37	42	61		97	52
3	2	4	6	10	5	7		13	7
4	10	19	9	14	9	13		28	15
Low level 1–2	42	78	48	76	55	80	0.88	145	78
High level 3–4	12	22	15	24	14	20		41	22
ASA									
1	9	18	4	7	24	35	0.002	37	21
2	30	61	43	70	33	48		106	59
3–4	10	20	14	23	12	17		36	20
NK	5		2		–			7	
Treatment									
Surgery (S) alone	22	41	30	48	23	33	0.02	75	40
S and RT primary	–	–	–	–	1	1		1	1
S and RT post-op	30	56	19	30	36	52		85	46
S and CT	–	–	3	5	1	1		4	2
S and RT post-op and CT	2	4	11	17	8	12		21	11
Surgery (S) alone	22	41	30	48	23	33	0.26	75	40
S and (RT or CT or RTCT)	32	59	33	52	46	67		111	60
Flap									
No	15	28	23	37	6	9	< 0.001	44	24
Soft	13	24	24	38	44	64		81	44
Composite****	26	48	16	25	19	28		61	33
Flap detail									
None	15	28	23	37	6	9	–	44	24
Radial	10	19	7	11	35	51		52	28
ALT	6	11	11	17	10	14		27	15
Lat dorsi	3	6	2	3	3	4		8	4
Rectus abdom	1	2	4	6	–	–		5	3
Buccal fat pad	–	–	1	2	1	1		2	1
DCIA	9	17	11	17	2	3		22	12
Scapula	5	9	1	2	7	10		13	7
Fibula	4	7	2	3	5	7		11	6
Lat dorsi and DCIA	1	2	–	–	–	–		1	1
Lat dorsi and scap	–	–	1	2	–	–		1	1
LOS (days)									
≤ 7	8	18	20	36	26	38	< 0.001	54	32
8–14	11	24	20	36	32	46		63	37
≥ 15	26	58	15	27	11	16		52	31
NK	9		8		–			17	
Median (IQR)	15 (9–22)		10 (4–15)		9 (7–13)		0.001	10 (7–16)	

*RT* radiotherapy, *CT* chemotherapy

*Fishers exact test, apart from use of Kruskal–Wallis test for Age and LOS. NK (not known) groups excluded

**24 of the 130 pN0 were based on clinical grounds only—no neck access with 11 of 41, 3 of 39 and 10 of 50 across the time periods

***Across time periods the 26 non-SCC comprised: acinic cell (0,0,1), adenocarcinoma (0,3,6), ameloblastoma (0,4,0), melanoma (0,2,2), Merkel cell (0,1,0),mucoepidermoid (1,1,0), non-keratinising Schneiderian (0,0,1), Sarcoma (0,0,1), Sinonasal undifferentiated (0,1,0) verrucous (0,0,2)

****For 2 patients, both soft and composite flaps were used (Lat dorsi and scapula, Lat dorsi and DCIA), coded here under composite

No oral rehabilitation was provided for 43% (78/183), while implant supported prostheses were provided for 36% (65/183), non-implant prostheses for 31% (39/183) and both types for 1 patient. There were significant changes over time (Table 2) with implant prostheses provision rising from 22% for 1994–2009 and 29% for 2010–2015 to 52% for 2016–2020; correspondingly, the use of non-implant support declined from 39 to 18% and 12%, respectively. Most implant support was by bridge or retained obturator while most non-Implant support was by obturator. Over time, there was a notable rise after 2015 in the use of a zygomatic implant supported implant bridge and a reduction in other forms of implant support; there was also a steady decline across the study period in the use of non-implant obturator support. Most (91%) implants before 2010 were performed after the primary surgery, while most after 2010 were performed at the same time as initial surgery (67% for 2010–2015 and 92% for 2016–2020). Patients significantly less likely to have rehabilitation (Table 3) were those with high-level Browns classification (63% level 3–4 vs 37% level 1–2), those treated by surgery with radiotherapy and chemotherapy (76%) compared with 33% for surgery alone and 40% for surgery with either radiotherapy or chemotherapy but not both, those with flap reconstruction (60% composite, 43% soft, 18% no flap), younger patients (57% if < 65 years, 40% if 65–74 years and 29% if ≥ 75 years), and those with longer LOS (28% if ≤ 7 days, 43% if 8–14 days and 57% if ≥ 15 days). For those that had rehabilitation, there were no notable associations amongst the casemix variables with whether the rehabilitation was implant or non-implant supported (Table 3).

**Table 2:** Rehabilitation, 1994–2020

	1994–2009			2010–2015			2016–2020			*P* value*	Total	
	*N*	%		*N*	%		*N*	%			*N*	%
All patients	54	100		63	100		69	100			186	100
Rehabilitation (prostheses)												
None provided	20		39	33		52	25		36	< 0.001	78	43
Implant	11		22	18		29	36		52		65	36
Non-implant	20		39	11		17	8		12		39	21
Both types	–			1		2	–				1	1
NK	3			–			–				3	
Rehabilitation detail												
None provided	20		39	33		52	25		36		78	43
Implant:												
Bridge	1			5			30				36	
Retained obturator	6			9			1				16	
Retained overdenture	2			3			1				6	
Crown	–			–			1				1	
Nasal/facial	–			1			2				3	
Orbital and overdenture	1			–			–				1	
Bridge and orbital	1			–			1				2	
Non-implant:												
Obturator	16			9			3				28	
Denture	–			1			3				4	
Orbital	4			1			1				6	
Direct and indirect restoration	–			–			1				1	
Other:												
Implant bridge and non-implant obturator	–			1			–				1	
Months from operation to implant												
Known for	11			18			36				65	
Median (IQR)	22 (10–31)			0 (0–4)			0 (0–0)			< 0.001	0 (0–2)	
Same day as operation	1		9	12		67	33		92	< 0.001	46	71

*Fishers exact test, apart from use of Kruskal–Wallis test for months between operation and implant

**Table 3:** Casemix and rehabilitation

	Total	No rehab		Implant rehab**		Non-implant rehab		*P* value* (no rehab vs yes)	*P* value* Implant vs non-implant (if rehab)
		*N*	%	*N*	%	*N*	%		
All patients	183	78	43	66	36	39	21		
Gender									
Male	91	48	53	28	31	15	16	0.007	0.84
Female	92	30	33	38	41	24	26		
Age (operation)									
< 65	68	39	57	19	28	10	15	0.004	0.19
65–74	52	21	40	23	44	8	15		
≥ 75	63	18	29	24	38	21	33		
Site									
Alveolus	102	40	39	38	37	24	24	0.12	0.12
Hard palate	35	12	34	17	49	6	17		
Max sinus	33	20	61	5	15	8	24		
Nasal	13	6	46	6	46	1	8		
Overall P stage									
Early (1–2)	39	12	31	16	41	11	28	0.07	0.64
Late (3–4)	130	62	48	45	35	23	18		
NK	14	4		5		5			
Diagnosis									
SCC	157	68	43	57	36	32	20	0.68	0.58
Not SCC	26	10	38	9	35	7	27		
Browns classification									
1	47	17	36	16	34	14	30	0.02	0.02
2	96	36	38	43	45	17	18		
3	13	10	77	2	15	1	8		
4	27	15	56	5	19	7	26		
Low level 1–2	143	53	37	59	41	31	22	0.006	0.10
High level 3–4	40	25	63	7	18	8	20		
ASA									
1	35	20	57	12	34	3	9	0.13	0.36
2	106	40	38	39	37	27	25		
3–4	35	14	40	13	37	8	23		
NK	7	4		2		1			
Treatment									
Surgery (S) alone	75	25	33	28	37	22	29	< 0.001	0.14
S and RT primary	1	1	100	–		–			
S and RT post-op	82	32	39	33	40	17	21		
S and CT	4	4	100	–		–			
S and RT post-op and CT	21	16	76	5	24	–			
Surgery (S) alone	75	25	33	28	37	22	29	0.05	0.23
S and (RT or CT or RTCT)	108	53	49	38	35	17	16		
Flap									
None	44	8	18	17	39	19	43	< 0.001	0.05
Soft	81	35	43	34	42	12	15		
Composite	58	35	60	15	26	8	14		
LOS (days)									
≤ 7	54	15	28	23	43	16	30	0.01	0.02
8–14	63	27	43	29	46	7	11		
≥ 15	49	28	57	10	20	11	22		
NK	17	8		4		5			

*RT* radiotherapy, *CT* chemotherapy

*Fishers exact test, excluding NK (not known) where applicable

**One case supported by both implant and non-implant prostheses was grouped under implant

***22 of the 127 pN0 were based on clinical grounds only—no neck access with 5 of 78, 9 of 66 and 8 of 39 across the three groups

The use of reconstructive flaps (76% overall) was associated significantly with many of the casemix variables (Table 4). Flap use was higher for patients with maxillary sinus tumours, more advanced tumour staging, high-level Browns classification, treated with radiotherapy and/or chemotherapy in addition to the surgery, and for patients with longer LOS. For those that had flap reconstructions, the most notable indicators towards having composite rather than soft flaps were younger age (62% 34/55 for < 65 years, 31% 27/87 for ≥ 65 years), more advanced tumour staging (50% 58/117 for late 3–4 overall stage, 5% (1/21) for earlier staging), involvement of radiotherapy and/or chemotherapy treatment (52% 53/101 with, 20% 8/41 without) and longer LOS (47% 51/108 ≥ 8 days, 17% 4/23 ≤ 7 days). For those without a flap, the median (IQR) LOS was 3 (1–7) days, compared to 11 (7–18) days with soft flap and 14 (10–17) days with composite flap, Kruskal–Wallis test *P* < 0.001 comparing all 3 groups and *P* = 0.02 (borderline sign) comparing soft flap with composite flap.

**Table 4:** Casemix and type of flap

	Total	No flap		Soft flap		Composite flap		*P* value* (no flap vs yes)	*P* value* Soft vs composite) (if flap)
		*N*	%	*N*	%	*N*	%		
All patients	186	44	24	81	44	61	33		
Gender									
Male	94	15	16	38	40	41	44	0.02	0.02
Female	92	29	32	43	47	20	22		
Age (operation)									
< 65	68	13	19	21	31	34	50	0.10	0.001
65–74	55	10	18	30	55	15	27		
≥ 75	63	21	33	30	48	12	19		
Site									
Alveolus	104	29	28	46	44	29	28	< 0.001	0.28
Hard palate	35	12	34	14	40	9	26		
Max sinus	34	–	–	18	53	16	47		
Nasal	13	3	23	3	23	7	54		
Overall P stage									
Early (1–2)	39	18	46	20	51	1	3	< 0.001	< 0.001
Late (3–4)	133	16	12	59	44	58	44		
NK	14	10		2		2			
Diagnosis									
SCC	160	34	21	71	44	55	34	0.08	0.79
Not SCC	26	10	38	10	38	6	23		
Browns classification									
1	48	25	52	17	35	6	13	< 0.001	0.05
2	97	19	20	47	48	31	32		
3	13	–	–	4	31	9	69		
4	28	–	–	13	46	15	54		
Low level 1–2	145	44	30	64	44	37	26	< 0.001	0.02
High level 3–4	41	–	–	17	41	24	59		
ASA									
1	37	3	8	20	54	14	38	0.03	0.89
2	106	29	27	43	41	34	32		
3–4	36	10	28	16	44	10	28		
NK	7	2		2		3			
Treatment									
Surgery (S) alone	75	34	45	33	44	8	11	< 0.001	0.002
S and RT primary	1	–	–	–	–	1	100		
S and RT post-op	85	9	11	37	44	39	46		
S and CT	4	–	–	2	50	2	50		
S and RT post-op and CT	21	1	5	9	43	11	52		
Surgery (S) alone	75	34	45	33	44	8	11	< 0.001	< 0.001
S and (RT or CT or RTCT)	111	10	9	48	43	53	48		
LOS (days)									
≤ 7	54	31	57	19	35	4	7	< 0.001	0.02
8–14	63	5	8	33	52	25	40		
≥ 15	52	2	4	24	46	26	50		
NK	17	6		5		6			

*RT* radiotherapy, *CT* chemotherapy

*Fishers exact test, excluding NK (not known) where applicable

**24 of the 130 pN0 were based on clinical grounds only—no neck access with 12 of 44, 7 of 81 and 5 of 61 across the three groups

**Fig. 1:**
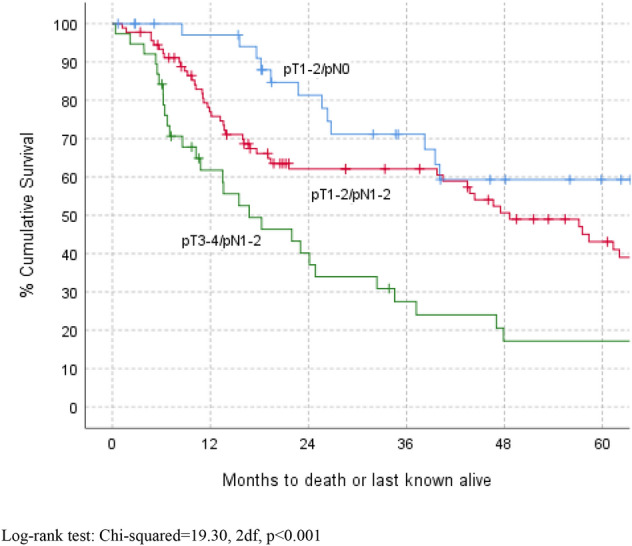
Kaplan–Meier survival by pTN stage. Log-rank test: Chi-squared = 19.30, 2df, *P* < 0.001

**Fig. 2:**
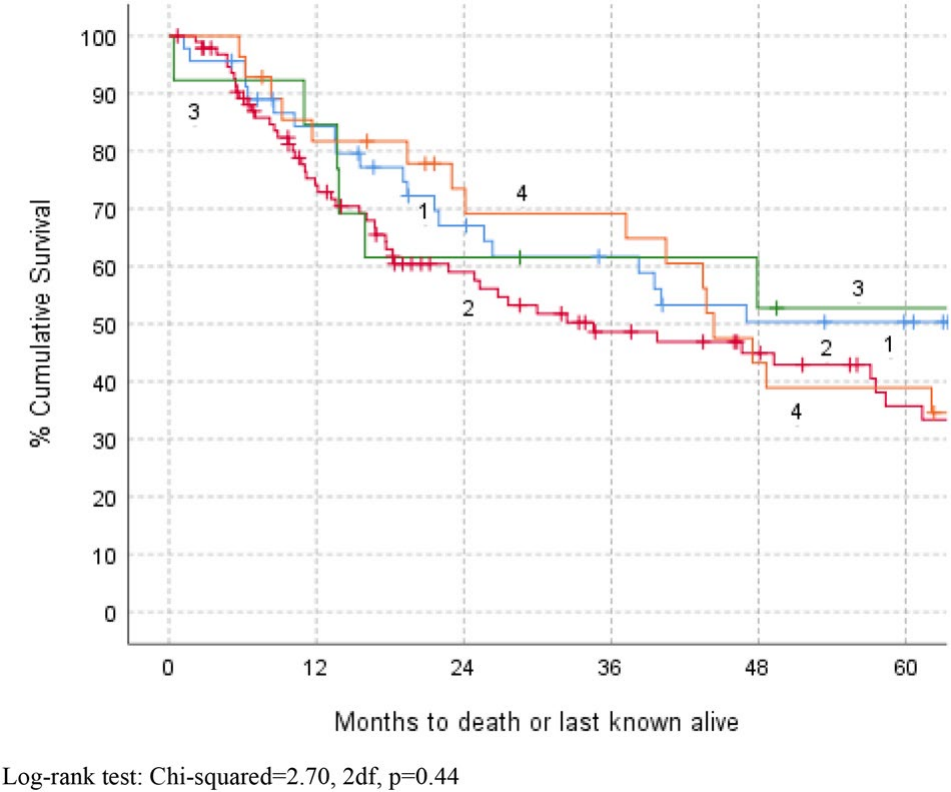
Kaplan–Meier survival by Browns classification (1–4). Log-rank test: Chi-squared = 2.70, 2df, *P* = 0.44

Overall survival after 24 months was 64% (SE 4%) and after 60 months was 42% (SE 4%). Casemix factors most strongly associated with better survival (Table 5) were less advanced tumour staging, rehabilitation support and a shorter LOS. Survival curves by pTN staging and by Browns classification are shown in Figs. 1 and 2 respectively. The overall recurrence rate was 40% (74/186), with little difference noted when analysed by Browns classification (Level 1: 40%, 19/48, Level 2: 40%, 39/97, Level 3: 38%, 5/13, Level 4: 39%, 11/28).

**Table 5:** Kaplan–Meier survival by casemix and rehabilitation

	Known for	12 months	24 months	36 months	48 month	60 months	*P* value*
All patients	183	79 (3)	64 (4)	56 (4)	46 (4)	42 (4)	
Time period							
1994–2009	54	81 (5)	64 (7)	57 (7)	47 (7)	41 (7)	0.71
2010–2015	60	70 (6)	63 (6)	58 (7)	46 (7)	43 (7)	
2016–2020	69	85 (5)	61 (7)	52 (8)	44 (9)	NA	
Gender							
Male	93	76 (5)	64 (5)	58 (5)	46 (6)	40 (6)	0.88
Female	90	81 (4)	63 (5)	55 (6)	47 (6)	43 (6)	
Age							
< 65	67	77 (5)	65 (6)	62 (6)	53 (7)	53 (7)	0.04
65–74	54	81 (5)	68 (7)	60 (7)	51 (8)	36 (8)	
≥ 75	62	78 (5)	58 (7)	48 (7)	35 (7)	32 (7)	
Site							
Alveolus	101	81 (4)	63 (5)	53 (5)	48 (6)	45 (6)	0.31
Hard palate	35	83 (6)	71 (8)	68 (8)	55 (9)	47 (9)	
Max sinus	34	76 (7)	63 (9)	59 (9)	39 (10)	32 (10)	
Nasal	13	49 (17)	49 (17)	49 (17)	33 (18)	17 (15)	
Overall P stage							
Early (1–2)	38	97 (3)	81 (7)	71 (8)	59 (9)	59 (9)	0.007
Later (3–4)	132	73 (4)	56 (5)	51 (5)	41 (5)	36 (5)	
Diagnosis							
SCC	158	79 (3)	62 (4)	54 (4)	44 (4)	40 (4)	0.63
No SCC	25	75 (9)	70 (10)	65 (10)	58 (11)	52 (12)	
Browns classification							
Low (1–2)	142	78 (4)	62 (4)	53 (5)	46 (5)	41 (5)	0.67
High (3–4)	41	83 (6)	70 (7)	64 (8)	47 (8)	44 (8)	
ASA							
1	37	87 (6)	56 (9)	52 (9)	36 (9)	36 (9)	0.97
2	103	76 (4)	67 (5)	57 (5)	49 (5)	43 (6)	
3–4	36	78 (7)	62 (9)	58 (9)	44 (10)	39 (10)	
Treatment							
Surgery (S) alone	73	84 (4)	73 (5)	67 (6)	59 (6)	53 (7)	0.02
S and (RT or CT or RTCT)	110	75 (4)	57 (5)	49 (5)	37 (5)	34 (5)	
Flap							
No	42	85 (6)	80 (6)	69 (7)	57 (8)	51 (8)	0.18**
Soft	81	77 (5)	60 (6)	55 (6)	43 (6)	41 (6)	
Composite	60	77 (6)	56 (7)	49 (7)	42 (7)	37 (7)	
LOS (days)							
≤ 7	53	89 (4)	79 (6)	70 (7)	57 (8)	53 (9)	0.004
8–14	63	71 (6)	64 (6)	53 (7)	41 (7)	38 (7)	
≥ 15	52	77 (6)	47 (7)	44 (7)	35 (7)	32 (7)	
Rehabilitation (Prostheses)							
None provided	75	70 (5)	47 (6)	41 (6)	26 (6)	26 (6)	0.001
Non-implant	39	87 (5)	71 (7)	62 (8)	52 (9)	45 (9)	
Implant	65	83 (5)	80 (5)	72 (6)	70 (7)	62 (8)	

*RT* radiotherapy, *CT* chemotherapy, *NA* not applicable

*Log-rank test, excluding NK (not known) where applicable

**P = 0.07 for flap (soft or composite) vs no flap

## Discussion

Controversy remains regarding the most appropriate reconstruction and oral rehabilitation for the post-oncology maxillary defect. As treatments become more refined over time, there is merit in reflecting back over several decades as this allows an opportunity to report changes in treatment strategies, survival and rehabilitation outcomes. Although the data are limited to one Institution, it represents a substantial series collated over many years. It is accepted that as a retrospective casenote review, there are various other aspects that are important which it has not been possible to report in this study, such as post-operative complications, donor site morbidity and health-related quality of life (HRQOL).

In our sample, Brown’s classification 1 and 2 predominate (78% of cases). In this relatively low-level defect, the main component of dysfunction in form and function is related to the teeth and the surgically created fistula between the oral cavity and the nose/maxillary sinus. The importance of oral function to HRQOL is well recognised [25] and issues of chewing, dental appearance, teeth, speech are amongst the commonest concerns [26]. Oral rehabilitation has a critical role in promoting optimal patient reported outcomes [25, 27]. The pathology, whether benign or malignant is an important consideration in terms of resection and reconstruction. In this case series, the focus has been on cancer and unsurprisingly the majority were squamous cell carcinoma. Over the time period of this study, there has been centralisation of the service [28] and this might account for variations in pathology where some earlier cases would have been managed in the surrounding hospitals and not referred. Resection for benign disease such as pleomorphic adenoma tends to be less destructive in relation to achieving margins and post-operative radiotherapy can be avoided. In our oncology cohort, over half of patients received post-operative radiotherapy and this is an important consideration in respect to treatment burden, and the scope for successful primary or secondary oral rehabilitation. Another essential aspect is the tumour stage and hence prognosis. Three quarters were pathological stage 3 or 4 and a quarter had cervical lymph node metastases. With advanced stage disease, the rates of failure over the first 2 years increase and there is a management imperative to help enable the patient to achieve the best possible HRQOL in the time remaining. In addition, specifically in this group, the burden of additional treatments should be minimised, for example, the need for multiple hospital visits for oral rehabilitation, further surgery, and modifications to the prosthesis. Ideally, there needs to be coordinated multi-disciplinary clinical review.

The series presented demonstrates that there is a definite place for obturation. Of the 45 patients, around one-third were implant retained and the remainder were managed without implants. In terms of the preferred methods of reconstruction, there has been a notable change over time. The vast majority of patients now have microvascular free tissue transfer. Furthermore, there has been less reliance on composite flaps to restore the defect with around half in last 5 years having radial free flaps. This has been accompanied by a reduction in hospital length of stay. The main driver for this change is the more frequent use of remotely anchored zygomatic implants to support the prosthesis [29–33]. With this technique, there is less reliance on bone transfer and an increasing focus on primary placement of zygomatic implants at the time of surgery. In terms of soft tissue, both the radial and the anterolateral thigh (ALT) free flaps have long pedicle lengths and this facilitates the ease of anastomosis in the neck. However, the ALT can be a bit too bulky for zygomatic implant perforation and subsequent fitting of the suprastructure. Despite the significant challenges, the ZIP flap technique provides full dental rehabilitation within 30 days of surgery and prior to radiotherapy if this is required, with excellent published patient reported outcomes [33]. There is a need for a bony free flap in the higher level maxillectomy defects [3], however, the Brown 3 and 4 defects are less common. Another factor which has influenced the change in practice is the recognition that in those patients in the past with relatively low-level defects and bony flap, the bone tended not to be used for oral rehabilitation and was not critical for restoration of facial profile. In addition, there has been concern regarding the placement of secondary implants in the irradiated field. Although optimal rehabilitation might not be achieved without the use of implant retention, their placement carries the risk of implant failure and osteoradionecrosis, and might confer only modest improvements in function and HRQOL [25, 32]. Another factor that limits delayed oral rehabilitation is that a proportion develops loco-regional failure and are not surviving after 2 years [17, 34–36]. It is encouraging that 64% in this cohort were alive after 24 months and 42% after 60 months. Tumour pathology is the main factor associated with survival factor but perhaps in a larger future sample it might be possible to tease out the relative risk of avoiding post-operative radiotherapy (de-escalation) in the low-level maxillectomy and report the benefit of this in terms of trade-off in treatment burden, side-effects and HRQOL.

There is still a place for composite free tissue alone, especially in the extensive resections, to provide adequate function and HROQOL, accepting that formal oral rehabilitation will not be achieved [19]. However, the emergence of zygomatic implants informs the previous algorithms for maxillary reconstruction [3–5, 10–15]. It is accepted that the utilisation of this approach relies on the experience and expertise of the clinician placing the implants and the maxillofacial prosthodontic team providing the prosthesis. This resource is available in our Institution and has allowed this approach to be routinely considered. Some units might wish to use computer-derived models and guides to assist with the placement of bone and implants at the time of primary surgery [37–39]. Whatever techniques are used to facilitate the accurate placement of the implants the imperative remains for these to be used for rehabilitation without undue delay.

In conclusion, there has been a change in the pattern on reconstruction of the maxillary defect over the last 3 decades. The majority now have free tissue reconstruction and there is a stronger emphasis on the incorporation of primary zygomatic implants and oral rehabilitation via early-loaded implant supported fixed bridge reconstruction. Although the prognosis is still relatively poor, early meaningful rehabilitation is feasible and the combination of zygomatic implants has changed the paradigm as to the optimal microvascular reconstruction.

## Declarations

The study complied with all aspects of ethical standards of clinical research.

## References

[1] Triana RJ, Uglesic V, Virag M, Varga SG, Knezevic P, Milenovic A, Aljinovic N, Murakami CS, Futran ND (2000). Microvascular free flap reconstructive options in patients with partial and total maxillectomy defects. Arch Facial Plast Surg.

[2] Mücke T, Hölzle F, Loeffelbein DJ, Ljubic A, Kesting M, Wolff KD, Mitchell DA (2011). Maxillary reconstruction using microvascular free flaps. Oral Surg Oral Med Oral Pathol Oral Radiol Endod.

[3] Brown JS, Rogers SN, McNally DN, Boyle M (2000). A modified classification for the maxillectomy defect. Head Neck.

[4] Cordeiro PG, Santamaria E (2000). A classification system and algorithm for reconstruction of maxillectomy and midfacial defects. Plast Reconstr Surg.

[5] Okay DJ, Genden E, Buchbinder D, Urken M (2001). Prosthodontic guidelines for surgical reconstruction of the maxilla: a classification system of defects. J Prosthet Dent.

[6] Santamaria E, Cordeiro PG (2006). Reconstruction of maxillectomy and midfacial defects with free tissue transfer. J Surg Oncol.

[7] Brown JS, Shaw RJ (2010). Reconstruction of the maxilla and midface: introducing a new classification. Lancet Oncol.

[8] Bidra AS, Jacob RF, Taylor TD (2012). Classification of maxillectomy defects: a systematic review and criteria necessary for a universal description. J Prosthet Dent.

[9] Akinmoladun VI, Dosumu OO, Olusanya AA, Ikusika OF (2013). Maxillectomy defects: a suggested classification scheme. Afr J Med Med Sci.

[10] Boyes-Varley JG, Howes DG, Davidge-Pitts KD, Brånemark I, McAlpine JA (2007). A protocol for maxillary reconstruction following oncology resection using zygomatic implants. Int J Prosthodont.

[11] Dalgorf D, Higgins K (2008). Reconstruction of the midface and maxilla. Curr Opin Otolaryngol Head Neck Surg.

[12] Cordeiro PG, Chen CM (2012). A 15-year review of midface reconstruction after total and subtotal maxillectomy: part I. Algorithm and outcomes. Plast Reconstr Surg.

[13] Hanasono MM, Silva AK, Yu P, Skoracki RJ (2013). A comprehensive algorithm for oncologic maxillary reconstruction. Plast Reconstr Surg.

[14] Costa H, Zenha H, Sequeira H, Coelho G, Gomes N, Pinto C, Martins J, Santos D, Andresen C (2015). Microsurgical reconstruction of the maxilla: algorithm and concepts. J Plast Reconstr Aesthet Surg.

[15] Eskander A, Kang SY, Teknos TN, Old MO (2017). Advances in midface reconstruction: beyond the reconstructive ladder. Curr Opin Otolaryngol Head Neck Surg.

[16] Rogers SN, Brown JS, Woolgar JA, Lowe D, Magennis P, Shaw RJ, Sutton D, Errington D, Vaughan D (2009). Survival following primary surgery for oral cancer. Oral Oncol.

[17] Sun Q, Zhang WB, Gao M, Yu S, Mao C, Guo CB, Yu GY, Peng X (2020). Does the Brown classification of maxillectomy defects have prognostic prediction for patients with oral cavity squamous cell carcinoma involving the maxilla?. Int J Oral Maxillofac Surg.

[18] Dos-Santos DM, de-Caxias FP, Bitencourt SB, Turcio KH, Pesqueira AA, Goiato MC (2018). Oral rehabilitation of patients after maxillectomy. A systematic review. Br J Oral Maxillofac Surg.

[19] Petrides GA, Hicks G, Dunn M, Froggatt C, Wallace C, Howes D, Leinkram D, Low TH, Ch'ng S, Wykes J, Palme CE, Clark JR (2021). Dentoalveolar outcomes in maxillary reconstruction: a retrospective review of 85 maxillectomy reconstructions. ANZ J Surg.

[20] National Clinical Coding Standards OPCS-4 (2017) Copyright © 2017 health and social care information centre. In: The health and social care information centre is a non-departmental body created by statute, also known as NHS Digital. http://systems.digital.nhs.uk/data/clinicalcoding

[21] Rogers SN, Lowe D, McNally D, Brown JS, Vaughan ED (2003). Health-related quality of life after maxillectomy: a comparison between prosthetic obturation and free flap. J Oral Maxillofac Surg.

[22] Brown JS, Bekiroglu F, Shaw RJ, Woolgar JA, Triantafyllou A, Rogers SN (2013). First report of elective selective neck dissection in the management of squamous cell carcinoma of the maxillary sinus. Br J Oral Maxillofac Surg.

[23] Mayhew D, Mendonca V, Murthy BVS (2019). A review of ASA physical status—historical perspectives and modern developments. Anaesthesia.

[24] Lydiatt WM, Patel SG, Osullivan B, Brandwein MS, Ridge JA, Migliacci JC, Loomis AM, Shah JP (2017). Head and Neck cancers-major changes in the American Joint Committee on cancer eighth edition cancer staging manual. CA Cancer J Clin.

[25] (2021) http://www.handle-on-qol.com/Index.aspx. Accessed 24 Dec 21

[26] Kanatas A, Ghazali N, Lowe D, Udberg M, Heseltine J, O'Mahony E, Rogers SN (2013). Issues patients would like to discuss at their review consultation: variation by early and late stage oral, oropharyngeal and laryngeal subsites. Eur Arch Otorhinolaryngol.

[27] Pace-Balzan A, Butterworth C, Lowe D, Rogers SN (2009). The responsiveness of the Liverpool oral rehabilitation questionnaire (LORQ): a pilot study. Int J Prosthodont.

[28] Hughes C, Homer J, Bradley P, Nutting C, Ness A, Persson M, Jeffreys M, Waylen A, Leary S, Thomas S (2012). An evaluation of current services available for people diagnosed with head and neck cancer in the UK (2009–2010). Clin Oncol (R Coll Radiol).

[29] Chrcanovic BR, Abreu MH (2013). Survival and complications of zygomatic implants: a systematic review. Oral Maxillofac Surg.

[30] Huang W, Wu Y, Zou D, Zhang Z, Zhang C, Sun J, Xu B, Zhang Z (2014). Long-term results for maxillary rehabilitation with dental implants after tumor resection. Clin Implant Dent Relat Res.

[31] Butterworth CJ, Rogers SN (2017). The zygomatic implant perforated (ZIP) flap: a new technique for combined surgical reconstruction and rapid fixed dental rehabilitation following low-level maxillectomy. Int J Implant Dent..

[32] Hackett S, El-Wazani B, Butterworth C (2021). Zygomatic implant-based rehabilitation for patients with maxillary and mid-facial oncology defects: a review. Oral Dis.

[33] Butterworth CJ, Lowe D, Rogers SN (2021). The Zygomatic Implant Perforated (ZIP) flap reconstructive technique for the management of low-level maxillary malignancy—clinical & patient related outcomes on 35 consecutively treated patients. Head Neck.

[34] Bhattacharyya N (2003). Factors affecting survival in maxillary sinus cancer. J Oral Maxillofac Surg.

[35] Lin HW, Bhattacharyya N (2009). Survival impact of nodal disease in hard palate and maxillary alveolus cancer. Laryngoscope.

[36] Hakim SG, Steller D, Sieg P, Rades D, Alsharif U (2020). Clinical course and survival in patients with squamous cell carcinoma of the maxillary alveolus and hard palate: results from a single-center prospective cohort. J Craniomaxillofac Surg.

[37] Rude K, Thygesen TH, Sørensen JA (2014). Reconstruction of the maxilla using a fibula graft and virtual planning techniques. BMJ Case Rep.

[38] Seikaly H, Idris S, Chuka R, Jeffery C, Dzioba A, Makki F, Logan H, O'Connell DA, Harris J, Ansari K, Biron V, Cote D, Osswald M, Nayar S, Wolfaardt J (2019). The alberta reconstructive technique: an occlusion-driven and digitally based jaw reconstruction. Laryngoscope.

[39] Tamaki A, Seim NB, Valentin S, Ozer E, Agrawal A, VanPutten M, Kang SY, Old MO (2020). The use of medical modeling in microvascular free tissue transfer reconstruction with osseointegrated implantation in complex midface defects. Oral Oncol.

